# Patient reported experiences and readmissions for people with diabetes-related foot disease admitted to public hospitals, New South Wales, Australia, 2019–2022

**DOI:** 10.1371/journal.pone.0314895

**Published:** 2024-12-05

**Authors:** Alexander C. R. Burnett, Jennifer Williamson, Aedan G. K. Roberts, Sadaf Marashi-Pour, Liz Hay

**Affiliations:** 1 Centre for Epidemiology and Evidence, New South Wales Department of Health, St Leonards, NSW, Australia; 2 Economics and Evaluation Unit, Strategic Reform and Planning, New South Wales Department of Health, St Leonards, NSW, Australia; 3 Bureau of Health Information, Sydney, NSW, Australia; Taipei Medical University, TAIWAN

## Abstract

**Objective:**

Patient reported measures of hospital care are known predictors of readmission, even after accounting for risk related to age and comorbidities. This study aimed to determine the association between patient experience of diabetes-related foot disease (DFD) hospital care and unplanned hospital readmission, with a primary focus on DFD-related readmissions and a secondary focus on all-cause readmissions.

**Methods:**

A retrospective longitudinal cohort study was conducted by linking NSW Adult Admitted Patient Survey data with administrative hospital data for persons hospitalised with DFD identified through diagnostic and/or procedure codes. Univariable and multivariable shared-frailty Cox regression models were used to examine the association between key aspects of patient experiences and 90-days unplanned hospital readmission over the period 2019–2022.

**Results:**

Overall, 3,173 DFD patients were included. Ninety-day readmission rates for respondents with DFD were 9% for DFD-related readmissions and 16% for all-cause readmissions. Adults with DFD who could not understand explanations offered by health professionals were at increased risk of DFD-related readmission compared to those who could always understand (Hazard ratio (HR) 2.43, CI: 1.47–4.00), as well as patients who did not feel well enough to leave hospital at discharge (HR 1.93, CI: 1.41–2.64) or reported the care received was not well organised (HR 2.24, 1.45–3.47). Patients reporting that they did not receive enough information regarding their condition, treatment, or how to manage care at home were found to have a DFD-related readmission risk that was 1.5 to 1.8-times greater than those who did. Similar patterns were observed for all-cause readmissions, albeit with generally smaller effect sizes.

**Conclusions:**

The findings highlight that elements of care related to communication, coordination, and involvement in decision making may influence unplanned readmission rates for patients with chronic conditions, such as DFD. The impact appears to be more pronounced for DFD-related readmissions compared to all-cause readmissions.

## Introduction

Diabetes mellitus is a complex group of metabolic disorders characterised by high blood glucose levels due to an absolute or relative deficit in insulin production or action [1]. Globally, the prevalence of diabetes has increased significantly over the past three decades, with an estimated 537 million adults (10% of the global population) living with diabetes in 2021, and a continuous rise in diabetes prevalence expected [2]. Undiagnosed, untreated, or poorly controlled, diabetes can result in chronic microvascular (peripheral neuropathy, diabetic nephropathy, retinopathy) and macrovascular (cardiovascular, cerebrovascular and peripheral vascular) complications [3], all of which are associated with increased morbidity and mortality [4].

Diabetes-related foot disease (DFD) is recognised as a leading cause of hospital admissions, amputations, disability, and health care costs globally [5–8]. DFD is defined by the International Working Group on the Diabetic Foot as ulceration, infection, or destruction of foot tissues in people with diabetes, typically accompanied by neuropathy and/or peripheral artery disease [9]. In New South Wales (NSW), Australia, the treatment of DFD accounted for 35,000 hospitalisations and an inpatient cost of nearly $330 million in 2014–15 [10]. Notably, approximately 30,500 of these hospitalisations and 80% of the cost were dedicated to the treatment of infections and ulcers [10]. Despite intensive treatment, such as wound care, off-loading, revascularisation, and glycaemic control, diabetes-related foot ulcer (DFU) recurrence is common, with 40% of ulcers reoccur within 1 year following wound closure and 60% within 3 years [5]. DFUs are also very vulnerable to infection, with 40% of ulcers developing an infection [11] and around 20% of moderate to severe diabetic foot infections resulting in a limb amputation [5]. Given the significant burden of DFD in NSW, analysing factors associated with readmission is not only clinically beneficial but may help reduce the increasing costs to the health system.

A recent systematic review and meta-analysis of risk factors for DFD readmission to hospital showed 30-day readmission risk was associated with female gender, peripheral neuropathy, lack of private health insurance, and coronary artery disease [12]. Whilst these risk factors may not be modifiable, exploring patients’ views and experiences of DFD-related care may help identify factors associated with unplanned and potentially preventable readmissions. Increasingly, attention has focused on the association between patient-reported experiences of care and readmissions [13]. There have been several studies which have reported that patient experience scores were more predictive of hospital readmission than clinical performance measures, such as the health status of patients [14–18]. In a recent Australian study among adults with COPD or CHF, patients who offered the most negative ratings for overall care, communication, organisation, and their preparedness for discharge were at a 1.5 to more than 2 times higher risk of readmission within 90-days [19]. This finding is supported by a larger Canadian study of unplanned all-cause readmissions between 43 and 365 days post-discharge where patients who self-reported that they were not involved in care decisions or hadn’t received written documentation post-discharge were 1.3- and 1.2-times more likely to be readmitted, respectively [20]. Such findings support that delivering value-based healthcare which improves overall patient experiences may progressively reduce the risk of adults with chronic conditions, such as DFD, returning to acute care.

Therefore, the aim of this study was to determine the association between patient experience of DFD-related care and unplanned hospital readmission within 90-days through the linkage of de-identified survey and hospital administrative data. We hypothesised that there would be a significant association between the quality of patient experience during their index admission for DFD care and the likelihood of unplanned readmissions within a 90-day period.

## Methods

### Setting

New South Wales (NSW) is Australia’s most populated state, with an estimated residential population of 8.2 million people in 2022 [21]. There are more than 220 public hospitals and health services in NSW which provide free health care to Australian citizens and permanent residents [22]. Services provided at NSW public hospitals include emergency care, elective and emergency surgery, medical treatment, maternity services, and rehabilitation programs.

In 2017, the Leading Better Value Care (LBVC) program was established as a NSW public health system priority to improve outcomes and experiences for people with specific conditions, including DFD, through a series of targeted evidence-based clinical initiatives [23]. The NSW Ministry of Health requested that the Bureau of Health Information conduct targeted oversampling of specific patient cohorts to support LBVC initiatives.

To provide further insights for patients’ outcomes and experience, the NSW Ministry of Health established the Register of Outcomes, Value, and Experience (ROVE) under the NSW Public Health Act 2010 to support the ongoing monitoring, evaluation, and assessment of the LBVC program by linking all admissions for patients included in LBVC cohorts with inpatient survey responses for persons consenting to link their patient survey data to other health datasets [23, 24]. The ROVE data linkage includes subsets of the Admitted Patient Data Collection (APDC), Registry of Births, Deaths, and Marriages (RBDM), and the NSW Patient Survey Program [24].

### Study design

This retrospective longitudinal cohort study was conducted by linking NSW Adult Admitted Patient Survey (AAPS) data for persons hospitalised with DFD identified through primary and/or secondary diagnostic and/or procedure codes (S1 Appendix), with APDC and RBDM data. Data linkage was performed by the NSW Centre for Health Record Linkage (CHeReL), which carries out probabilistic linkage of health-related data in accordance with NSW ethical, legal, privacy, and confidentiality requirements [24].

### Recruitment

All adults who have DFD admitted for acute care or rehabilitation from January to March 2019, May to July 2020 and 2021, or April to June 2022 were invited to complete the survey two to three months post discharge [23, 25–27]. The survey was followed up by two reminders to encourage participation. Surveys could be completed online or in paper format returned via mail by the patient. Clear instructions were provided in the survey to ensure that the responses were specific to certain hospital stays. These instructions clearly specified the hospital and date for which participants were asked to rate their experiences. Participants were eligible to receive a survey each year, however, only 107 people did so.

As per the AAPS inclusion criteria, patients under 18 years of age, records that do not have an episode of care type, patients who died during their hospital admission, patients receiving acute and post-acute care services, patients who are not receiving acute or rehabilitation care in hospital, patients who were admitted to a psychiatric unit during the hospital stay, patients admitted for same-day dialysis, patients who stayed for less than three hours, and same-day patients transferred to another hospital were excluded [23, 25–27].

Survey data for DFD patients was linked to corresponding APDC index admissions and unplanned readmissions using a de-identified unique project-specific Project Person Number provided by the CHeReL.

### Classification of admission and readmission

Patients were excluded from the study if the index separation status was ‘discharged at own risk’ or ‘transferred to palliative care’, where the index admission of interest was within 90-days of a previous discharge with DFD, or where a primary and/or secondary diagnosis code indicated that the patient was COVID positive. Transfers were considered and multiple, contiguous hospitalisations were considered as a single period of care [28].

A 90-day lookahead was used to identify all hospitalisation and mortality data following discharge for the index admission to detect all-cause readmissions and DFD-related readmissions (identified through primary and secondary diagnostic and/or procedure codes) to an NSW public or private hospital.

Readmissions were defined as acute and unplanned (i.e., emergency) returns to care to a NSW public or private hospital 1 or more days following discharge from an index admission. In cases where there were multiple unplanned readmissions within the relevant period following the initial admission, only the first subsequent readmission was considered. Acute non-emergency returns to care were not considered as care received could be related to planned follow-up care for to the index admission.

### Patient experience measures

Patient experience questions were prioritised based on key themes known to be important to patients, accreditation, and clinical outcomes, specifically compassion, respect, kindness, clear communication, involvement in decision making, and preparedness for discharge [29–31]. Survey questions were selected based on previous studies of patient experiences associated with readmissions to NSW hospitals [19, 32]. Survey questions assessed are included in Table 1.

**Table 1:** Diabetes-related foot disease patient reported experiences and outcomes assessed for association with readmission to hospital within 90-days, by readmission type, 2019–2022.

	DFD-related readmissions	All-cause readmissions
**Overall ratings of care and self-reported outcomes**		
Overall, how would you rate the care you received while in hospital?	✓	✓
Overall, how would you rate the doctors who treated you?	*	*
Overall, how would you rate the nurses who treated you?	*	✓
Did the care and treatment received in hospital help you?	✓	✓
If asked about your hospital experience by friends and family how would you respond?	✓	✓
**Compassion, respect, and kindness**		
Did you feel you were treated with respect and dignity while you were in the hospital?	*	✓
**Effective communication and clear communication**		
How much information about your condition or treatment was given to your family, carer or someone close to you?	*	✓
During your stay in hospital, how much information about your condition or treatment was given to you?	✓	✓
Did the health professionals explain things in a way you could understand?	✓	✓
Did hospital staff take your family and home situation into account when planning your discharge?	NS	*
Thinking about when you left hospital, were you given enough information about how to manage your care at home?	✓	✓
Did hospital staff tell you who to contact if you were worried about your condition or treatment after you left hospital?	NS	NS
**Involvement in decision-making**		
Did you feel involved in decisions about your discharge from hospital?	NS	NS
At the time you were discharged, did you feel that you were well enough to leave the hospital?	✓	✓
**Timely and coordinated care**		
How would you rate how well the health professionals worked together?	*	✓
How well organised was the care you received in hospital?	✓	✓
Thinking about when you left hospital, were adequate arrangements made by the hospital for any services you needed?	*	*
Did the hospital provide you with a document summarising the care you received in hospital (e.g., a copy of the letter to your GP or a discharge summary)?	✓	✓

Notes: ✓ indicates the overall p-value was statistically significant; * indicates although the overall p-value was not statistically significant, there were categories associated with readmission; NS indicates the association was not statistically significant.

### Demographic and clinical measures

Demographic characteristics were collected from both survey and linked admitted patient data. From the AAPS survey, language spoken at home, education level, and time of survey data were measured as categorical data. The linked admitted patient data provided sex, marital status, residential remoteness, and socio-economic status were measured as categorical data. Age was obtained from the linked admitted patient data and measured as a continuous variable and presented in years. Residential Statistical Area 2 (SA2) codes from the linked admitted patient data were used to classify remoteness levels according to the 2011 Australian Statistical Geographical Standard [33] and socio-economic status according to the 2011 Index of Relative Socio-economic Disadvantage (IRSD) quintiles [34]. Remoteness levels for outer regional, remote, and very remote categories were combined due to low numbers.

Clinical characteristics were measured from linked admitted patient data with a 12-month lookback period. Charlson comorbidity score [35], obesity status [36], and smoking status [36] were measured as categorical data from the linked admitted patient data. For patient characteristics and outcomes, categorical and count variables were presented as proportion of the total cohort, while continuous variables were presented as mean with standard deviation.

### Data analysis

Statistical analyses were carried out in SAS 9.4 [37], and a two-sided p value of <0.05 was taken as significant for all analyses. Univariable and multivariable shared-frailty Cox regression analyses, accounting for hospital cluster-specific random effects, were used to examine the association between key aspects of patient experience and unplanned hospital readmission with dates censored at 90-days after discharge. As death was one of the exclusion criteria, there was no competing risk of death. Hence, there was no requirement to utilise competing risks regression models. Separate models were developed for each of the key survey questions and each readmission group (i.e., all cause and DFD-related). Missing or inapplicable survey question responses were excluded. The prevalence of missing responses ranged from 1% to 3%.

Covariates included sociodemographic and clinical health characteristics obtained from both the patient survey and prior 12-months of administrative data to risk-adjust results [19, 38]. For each of the key survey questions, a backward selection approach was used to build the multivariable models. Sociodemographic and clinical variables with a *p*-value of <0.2 in the univariable analysis were considered for inclusion in the multivariable models, and variables with a two-sided *p*-value of <0.05 were retained in the final models. Multicollinearity was tested using the Variance Inflation Factor (VIF), and none of the variables included in the backward selection process were found to have high multicollinearity, ensuring that it did not impact the model estimates. The effect of excluding variables from the models on other coefficients was also evaluated. Variables not eligible for inclusion in the backward selection approach were then added individually and retained in the final models where *p* <0.05.

The proportional-hazards assumption was tested based on Schoenfeld residuals and it was not violated. Sensitivity analyses were performed using random effect logistic regression models, forcing age and sex into the models when not significant, which showed consistent results with the main analyses.

### Ethics

This study used data from the NSW Register of Outcomes, Value and Experience (ROVE), which was established under the Public Health Act 2010. As the project conformed to the standards established by the act, ethics committee approval was not necessary.

## Results

A total of 4,247 DFD patients were initially included in the study. Approximately 93% of the 2019–2022 survey data were linked with a corresponding acute DFD index hospitalisation, forming an initial cohort of 3,959 patients. After applying the exclusion criteria, 3,173 (80%) patients were included in the final analyses (S2 Appendix).

Of these, 297 (9%) had an unplanned emergency DFD-related readmission within 90-days of discharge, while 520 (16%) had an all-cause unplanned emergency readmission. Response rates and consent to data linkage varied annually with 30% of adults with DFD completing the survey and 82% consenting to data linkage in 2022 (34%, 85% in 2021, 36%, 79% in 2020 and 40%, 75% in 2019) [23, 25–27].

The respondents to the survey were compared with the general population admitted to NSW public hospitals with DFD in terms of age and sex. It was found that survey respondents were more likely to be male (65% vs 61%) and older, with a mean age of 74.0 years (SD = 10.7) compared to 67 years (SD = 18.7) for the general population. However, since the primary object of the study was to explore associations while adjusting for risk factors, including those typically used for sampling weights, the samples were not weighted.

The socio-demographic and clinical profiles of respondents are provided in Table 2. DFD-related readmissions were associated with Charlson comorbidity score, time of survey, and smoking. In comparison, all cause readmissions within 90-days were associated with Charlson comorbidity score, time of survey, age group, marital status, smoking and obesity.

**Table 2:** Demographic and clinical characteristics for DFD patients, 2019–2022.

			DFD-related readmissions		All-cause readmissions	
		Patients n (%)	Readmissions n (%)	p-value	Readmissions n (%)	p-value
**Total**		3173 (100)	297 (100)		520 (100)	
**Age, mean (SD)**		74.0 (11)	74.3 (11)	0.615	74.7 (11)	0.103
**Age group**						
	≤ 55 years	174 (5)	22 (7)		36 (7)	
	55–64 years	402 (13)	32 (11)		54 (10)	
	65–74 years	1005 (32)	87 (29)		149 (29)	
	75+ years	1592 (50)	156 (53)	0.252	281 (54)	0.034
**Sex**						
	Male	2075 (65)	200 (67)		337 (65)	
	Female	1098 (35)	97 (33)	0.459	183 (35)	0.758
**Marital status**						
	Married/de facto	1837 (58)	168 (57)		281 (54)	
	Never married	332 (10)	28 (9)		51 (10)	
	Divorced/separated	448 (14)	49 (17)		78 (15)	
	Widowed	53 (17)	52 (18)		109 (21)	
	Missing	19 (1)	0 (0)	0.437	1 (<1)	0.042
**Language spoken at home**						
	English	2764 (87)	258 (87)		450 (87)	
	Other	366 (12)	35 (12)		62 (12)	
	Missing	43 (1)	4 (1)	0.990	8 (2)	0.879
**Education**						
	Less than Y12	1429 (45)	135 (45)		231 (44)	
	Y12	445 (14)	44 (15)		68 (13)	
	Certificate	802 (25)	68 (23)		129 (25)	
	University	339 (13)	37 (12)		71(14)	
	Missing	98 (3)	13 (4)	0.617	21 (4)	0.575
**Remoteness level**						
	Major city	2031 (64)	204 (69)		347 (67)	
	Inner regional	855 (27)	68 (23)		133 (26)	
	Outer regional or remote	243 (8)	24 (8)		39 (8)	
	Missing	34 (1)	1 (<1)	0.162	1 (<1)	0.118
**IRSD SEIFA quintile**						
	1: Most disadvantaged	892 (28)	95 (32)		154 (30)	
	2	638 (20)	62 (21)		109 (21)	
	3	654 (21)	65 (22)		115 (22)	
	4	551 (17)	42 (14)		87 (17)	
	5: Least disadvantaged	404 (13)	32 (11)		54 (10)	
	Missing	34 (1)	1 (<1)	0.244	1 (<1)	0.120
**Comorbidity score**						
	0	1879 (59)	132 (44)		232 (45)	
	1	445 (14)	53 (18)		90 (17)	
	2	524 (17)	69 (23)		112 (22)	
	3+	325 (10)	43 (14)	<0.001	86 (17)	<0.001
**Time of survey**						
	2019	744 (23)	41 (14)		81 (16)	
	2020	876 (28)	95 (32)		169 (33)	
	2021	792 (25)	88 (30)		141 (27)	
	2022	761 (24)	73 (25)	<0.001	129 (25)	<0.001
**Smoking**						
	No	2859 (90)	255 (86)		453 (87)	
	Yes	314 (10)	42 (14)	0.010	67 (13)	0.013
**Obesity**						
	No	3108 (98)	288 (97)		503 (97)	
	Yes	65 (2)	9 (3)	0.210	17 (3)	0.032

In terms of annual admissions to hospital, 761 adult DFD respondents in 2022 (i.e., most recent year) had a total of 2311 acute periods of care (i.e., admissions, excluding transfers), with nearly 1 in 2 (49%) admissions principally for DFD. Throughout the year, approximately 2 in 5 had one acute admission (41%), 1 in 4 had two acute admissions (28%), and 1 in 3 had three or more acute admissions (31%). Similar patterns of annual hospital admissions were observed for other years (i.e., 2019–2021).

Overall ratings of care among adults with DFD were found to be associated with DFD-related readmissions within a 90-day period (Table 3). Respondents who would express a critical view of their hospital experience when discussing it with friends and family had an 85% (HR 1.85, CI: 1.21–2.82) higher DFD-related readmission risk than those who would speak highly of their hospital experience. Respondents who reported that their overall care as ‘poor or very poor’ (HR 2.14, CI: 1.22–3.76) or ‘neither good nor poor’ (HR 1.72, CI: 1.10–2.67) had a higher DFD-related readmission risk compared to those who reported the care provided was ‘very good’. Those reporting that the care and treatment they received in the hospital did not help at all had almost twice the DFD-related readmission risk (HR 1.96, CI: 1.21–3.17) compared to those who reported that it ’definitely helped’.

**Table 3:** Risk-adjusted statistically significant patient experiences associated with 90-day DFD-related readmissions for adults with DFD, 2019–2022.

			Patients (n)	Readmission (%)	Hazard ratio	p-value	95% CI		Overall p-value
Total			3173	9					
*Overall ratings of care and self-reported outcomes*									
Overall, how would you rate the care you received while in hospital?									
	Very good		2198	68	Ref				0.003
	Good		727	20	0.88	0.400	0.66	1.81	
	Neither good/poor		145	7	1.72	0.017	1.10	2.67	
	Poor/very poor		64	4	2.14	0.008	1.22	3.76	
Overall, how would you rate the doctors who treated you?									
	Very good		2239	67	Ref				0.071*
	Good		677	25	1.25	0.104	0.96	1.64	
	Neither good/poor		114	6	1.75	0.028	1.06	2.87	
	Poor/very poor		36	2	1.52	0.359	0.62	3.69	
Overall, how would you rate the nurses who treated you?									
	Very good		2414	76	Ref				0.155*
	Good		535	18	1.02	0.877	0.75	1.39	
	Neither good/poor		90	5	1.77	0.033	1.05	2.99	
	Poor/very poor		34	2	1.50	0.373	0.62	2.65	
Did the care and treatment received in hospital help you?									
	Yes definitely		2417	71	Ref				0.015
	Yes, some extent		604	22	1.21	0.173	0.92	1.61	
	No, not at all		106	6	1.96	0.006	1.21	3.17	
If asked about your hospital experience by friends and family how would you respond?									
	Speak highly		2490	73	Ref				0.006
	Neither highly/critical		481	19	1.34	0.053	1.00	1.80	
	Critical		150	8	1.85	0.005	1.21	2.82	
*Compassion*, *respect*, *and kindness*									
Did you feel you were treated with respect and dignity while you were in the hospital?									
	Yes, always		2720	85	Ref				0.114
	Yes, sometimes		310	12	1.23	0.248	0.86	1.76	
	No		51	3	1.85	0.071	0.95	3.62	
*Effective communication and clear communication*									
How much information about your condition or treatment was given to your family, carer or someone close to you?									
		Right amount	1860	77	Ref				0.211
		Not enough	393	22	1.32	0.078	0.97	1.79	
		Too much	16	1	1.06	0.933	0.26	4.30	
During your stay in hospital, how much information about your condition or treatment was given to you?									
		Right amount	2500	78	Ref				0.009
		Not enough	457	22	1.51	0.004	1.14	2.00	
		Too much	28	<1	0.37	0.325	0.05	2.67	
Did the health professionals explain things in a way you could understand?									
		Yes, always	2337	63	Ref				<0.001
		Yes, sometimes	654	31	1.71	<0.001	1.33	2.20	
		No	82	6	2.43	<0.001	1.47	4.00	
Thinking about when you left hospital, were you given enough information about how to manage your care at home?									
		Yes, completely	2096	66	Ref				0.006
		Yes, to some extent	599	23	1.26	0.115	0.95	1.67	
		No, not enough	188	11	1.83	0.002	1.24	2.70	
*Involvement in decision-making*									
At the time you were discharged, did you feel that you were well enough to leave the hospital?									
		Yes	2809	84	Ref				<0.001
		No	276	16	1.93	<0.001	1.41	2.64	
*Timely and coordinated care*									
How would you rate how well the health professionals worked together?									
		Very good	1879	57	Ref				0.064*
		Good	941	31	1.06	0.677	0.82	1.37	
		Neither good/poor	181	9	1.68	0.014	1.11	2.55	
		Poor/Very poor	62	3	1.55	0.202	0.79	3.03	
How well organised was the care you received in hospital?									
		Very well	2147	60	Ref				<0.001
		Fairly well	862	32	1.37	0.024	1.04	1.72	
		Not well	120	8	2.24	<0.001	1.45	3.47	
Thinking about when you left hospital, were adequate arrangements made by the hospital for any services you needed?									
		Yes, completely	1620	70	Ref				0.102*
		Yes, to some extent	394	23	1.36	0.045	1.01	1.85	
		No	200	7	0.90	0.678	0.55	1.47	
Did the hospital provide you with a document summarising the care you received in hospital (e.g., a copy of the letter to your GP or a discharge summary)?									
		Yes	2583	96	Ref				0.007
		No	277	4	0.45	0.007	0.25	0.80	

Note: Missing/not applicable responses were excluded. Multivariable models adjusted for Charlson Comorbidity score and survey time. Separate models were developed for each question; * indicates although the overall p-value was not statistically significant, there were categories associated with readmission.

Ineffective communication was identified as a significant factor associated with higher risk-adjusted DFD-related readmissions within 90-days. Specifically, respondents who reported that health professionals did not explain things in a way they could understand had more than twice the risk of readmission, compared to those who stated that health professionals ’always’ explained things clearly (HR 2.43, CI: 1.47–4.00). While DFD-related readmission risk was 71% (HR 1.71, CI: 1.33–2.20) higher among respondents who reported that health professionals ’sometimes’ explained things in a way they could understand. Respondents who reported that they did not receive enough information about their condition or treatment had a 51% (HR 1.51, CI: 1.14–2.00) higher DFD-related readmission risk compared to those who were given the ‘right amount’. The risk of DFD-related readmission was also higher among respondents who felt they did not receive enough home care management information, with a 83% (HR 1.83, CI: 1.24–2.70) higher readmission risk compared to those who were ‘completely’ informed.

Timely and coordinated care, along with active involvement in decision making, was also found to be associated with risk-adjusted readmissions within a 90-day period. Respondents who perceived the care they received in the hospital as ‘not well’ organised were at more than twice the risk of DFD-related readmission compared to those who reported that care was ‘very well’ organised (HR 2.24, CI: 1.45–3.47). A higher DFD-related readmission risk was also found among respondents who perceived that the care received was ‘fairly well’ organised (HR 1.37, CI: 1.04–1.72). Respondents who reported how well health professionals worked together as ‘neither good nor poor’ had a 68% (HR 1.68, CI: 1.11–2.55) higher DFD-related readmission risk compared to those who reported ‘very good’. Notably, respondents who stated that they were ‘not well’ enough to leave the hospital had almost twice the risk of DFD-related readmission compared to those who indicated they were ‘well’ enough (HR 1.93, CI: 1.41–2.64). Conversely, the absence of a document summarizing the care received in the hospital was associated with a reduced risk of readmission (HR 0.45, CI: 0.25–0.80).

Similar patterns were observed for all-cause readmissions within 90-days, albeit with generally smaller effect sizes and confidence intervals (Table 4). For instance, critical views of hospital experience were associated with an 82% increase in all-cause readmissions (HR 1.82, CI: 1.32–2.52) compared to an 85% increase in DFD-related readmissions. Ineffective communication showed a similar trend, with failure to understand explanations associated with an HR of 1.91 (CI: 1.26–2.90) for all-cause readmissions compared to an HR of 2.43 for DFD-related readmissions. The perception of poorly organised care was associated with an HR of 1.96 (CI: 1.38–2.78) for all-cause readmissions, slightly lower than the HR of 2.24 for DFD-related readmissions.

**Table 4:** Risk-adjusted statistically significant patient experiences associated with 90-day all cause readmissions for adults with DFD, 2019–2022.

			Patients (n)	Readmission (%)	Hazard ratio	p-value	95% CI		Overall p-value
Total			3173	16					
*Overall ratings of care and self-reported outcomes*									
Overall, how would you rate the care you received while in hospital?									
	Very good		2198	67	Ref				0.011
	Good		727	23	1.02	0.843	0.83	1.26	
	Neither good/poor		145	7	1.66	0.004	1.18	2.34	
	Poor/very poor		64	3	1.56	0.083	0.94	2.58	
Overall, how would you rate the doctors who treated you?									
	Very good		2239	69	Ref				0.096*
	Good		677	24	1.18	0.111	0.92	1.46	
	Neither good/poor		114	5	1.49	0.046	1.01	2.21	
	Poor/very poor		36	2	1.41	0.335	0.70	2.85	
Overall, how would you rate the nurses who treated you?									
	Very good		2414	73	Ref				0.016
	Good		535	20	1.25	0.050	1.00	1.55	
	Neither good/poor		90	5	1.76	0.006	1.18	2.65	
	Poor/very poor		34	1	1.25	0.560	0.59	2.65	
Did the care and treatment received in hospital help you?									
	Yes, definitely		2417	71	Ref				0.006
	Yes, some extent		604	25	1.37	0.003	1.12	1.68	
	No, not at all		106	4	1.38	0.142	0.90	2.13	
If asked about your hospital experience by friends and family how would you respond?									
	Speak highly		2490	75	Ref				0.001
	Neither highly/critical		481	17	1.17	0.194	0.92	1.47	
	Critical		150	8	1.82	<0.001	1.32	2.52	
*Compassion*, *respect*, *and kindness*									
Did you feel you were treated with respect and dignity while you were in the hospital?									
	Yes, always		2720	85	Ref				0.092
	Yes, sometimes		310	13	1.28	0.066	0.98	1.67	
	No		51	2	1.46	0.198	0.82	2.60	
*Effective communication and clear communication*									
How much information about your condition or treatment was given to your family, carer or someone close to you?									
		Right amount	1860	76	Ref				0.003
		Not enough	393	24	1.49	<0.001	1.18	1.89	
		Too much	16	1	1.02	0.969	0.33	3.20	
During your stay in hospital, how much information about your condition or treatment was given to you?									
		Right amount	2500	76	Ref				<0.001
		Not enough	457	23	1.69	<0.001	1.37	2.08	
		Too much	28	1	0.70	0.533	0.22	2.17	
Did the health professionals explain things in a way you could understand?									
		Yes, always	2337	66	Ref				<0.001
		Yes, sometimes	654	30	1.56	<0.001	1.28	1.89	
		No	82	5	1.91	0.002	1.26	2.90	
Did hospital staff take your family and home situation into account when planning your discharge?									
		Yes, completely	1987	72	Ref				0.035
		Yes, to some extent	475	20	1.16	0.224	0.92	1.46	
		No, not enough	167	8	1.52	0.015	1.09	2.13	
Thinking about when you left hospital, were you given enough information about how to manage your care at home?									
		Yes, completely	2096	66	Ref				<0.001
		Yes, to some extent	599	24	1.28	0.023	1.04	1.59	
		No, not enough	188	10	1.75	<0.001	1.28	2.38	
*Involvement in decision-making*									
At the time you were discharged, did you feel that you were well enough to leave the hospital?									
		Yes	2809	84	Ref				<0.001
		No	276	16	1.96	<0.001	1.54	2.49	
*Timely and coordinated care*									
How would you rate how well the health professionals worked together?									
		Very good	1879	56	Ref				0.006
		Good	941	32	1.14	0.176	0.94	1.39	
		Neither good/poor	181	9	1.64	0.003	1.19	2.27	
		Poor/Very poor	62	3	1.69	0.042	1.02	2.80	
How well organised was the care you received in hospital?									
		Very well	2147	61	Ref				<0.001
		Fairly well	862	32	1.30	0.007	1.07	1.57	
		Not well	120	7	1.96	<0.001	1.38	2.78	
Thinking about when you left hospital, were adequate arrangements made by the hospital for any services you needed?									
		Yes, completely	1620	70	Ref				0.167
		Yes, to some extent	394	21	1.26	0.061	0.66	1.60	
		No	200	8	1.00	0.982	0.70	1.44	
Did the hospital provide you with a document summarising the care you received in hospital (e.g., a copy of the letter to your GP or a discharge summary)?									
		Yes	2583	94	Ref				0.035
		No	277	6	0.68	0.035	0.57	0.97	

Note: Missing/not applicable responses were excluded. Multivariable models adjusted for Charlson Comorbidity score and survey time. Separate models were developed for each question; * indicates although the overall p-value was not statistically significant, there were categories associated with readmission.

## Discussion

Reducing the burden of diabetes-related foot disease is a major public health priority in NSW. Even with appropriate care, persistent DFD is associated with serious complications, such as infection, lower limb amputation, and even death [39–42]. To our knowledge, this is the first comprehensive state-wide analysis of patient self-reported experiences of care related to DFD, linking administrative hospital data to quantify the likelihood of unplanned DFD-related readmissions within 90-days post-discharge. We found that a poorer experience of inpatient care during the index admission is associated with a higher risk of DFD-related and all-cause readmission within 90-days. Our analysis showed that the quality of communication from health professionals and coordination of care were critical factors influencing this risk.

Whilst adults admitted to public hospitals in NSW for DFD related care mostly reported that health professionals always explained things in a way they could understand, many offered neutral or negative ratings. Respondents who could not understand explanations offered by health professionals were at more than twice the risk of DFD-related readmission. Moreover, more than 1 in 7 respondents also felt they were not given enough information regarding their care and treatment during their hospital stay or how to manage care at home, both of which were associated with increased readmission risk. Poor communication is one of the most cited underlying causes of patient complaints about the healthcare system [43]. In a qualitative meta-synthesis of perceptions and experiences of DFU-specific care, it was found that health professional to patient communication regarding their ulcer and foot care was generally considered to be poor [44]. One hypothesis for these findings is that the aetiology of DFU is complex and often difficult to communicate to patients [44], which can be further complicated by varying levels of health literacy [45]. Communication that is simple, clear, and individualised is integral to improving patient understanding of their condition and promoting engagement in effective self-care to reduce recurrence [45]. The development of DFU-specific communication models, such as the “Fragile Feet and Trivial Trauma” model [45], may offer potential for improving patient satisfaction with health professional communication during hospital DFD-related care. More generally, satisfaction with patient-health professional communication has been shown to be associated with better understanding of diabetes [46], self-care [47], and related health outcomes [48]. It is possible that by improving the communication of DFU aetiology patients may become more engaged in self-care, resulting in lower DFD readmission rates. However, at present, there is a lack of randomised controlled trials which have investigated whether DFU communication models improve patient outcomes and contribute to lower DFD readmission rates. Future research is required to assess the efficacy of such communication models in the Australian healthcare system.

There is strong evidence that the successful implementation of standardised care protocols and multidisciplinary teamwork can improve DFD-related outcomes. Recent systematic reviews and meta-analyses have demonstrated the effectiveness of these approaches [49–51]. Meta-analyses have shown that implementation of multidisciplinary care teams resulted in a 39–56% reduction in major amputation rates among adults with diabetes [49]. Organisational arrangements including multidisciplinary teams and care pathways have been associated with up to a 48% reduction in the risk of lower extremity amputations [50]. Structured diabetic foot care services have been found to significantly reduce major lower extremity amputation rates [51]. The findings of our study also support the essential role of well-organised and coordinated care in reducing the risk of hospital readmissions for patients with DFD. While most respondents reported that health professionals worked well together and that their care was well-organised–a possible reflection of the implementation of the Standards for High-Risk Foot Services (HRFS) [52] and the establishment of 29 hospital based HRFS in NSW [53]–those who experienced less organised care had almost double the risk of readmission. Whilst it is beyond the scope of this study to evaluate HRFS implementation across NSW, disparities suggest that the implementation may not be uniform across all patient experiences. Consequently, there may be opportunities to review the implementation of HRFS in-hospital care across NSW to improve patient experiences and minimise hospital readmissions in this vulnerable population.

Interestingly, patient-doctor and patient-nurse ratings revealed that those who reported their care as “neither good nor poor” had a higher risk of readmission. Whilst this is likely the result of very few patients endorsing that their care was “poor”, this finding still points to a potential area for improvement. It is important that patients feel satisfied with the care provided by their healthcare team, as this could influence their post-discharge behaviours, including adherence to treatments, thus potentially impacting readmission rates [54].

A somewhat counter-intuitive finding was that the absence of a document summarising the care received in the hospital was associated with a decreased risk of readmission. Previous research among the general inpatient population reported that patients who did not receive written information post discharge were at increased risk of readmission [20]. A possible explanation for our finding could be that DFD patients who did not receive such documentation may have received more verbal explanations, or perhaps more personalized and detailed care that was not captured in a standardized document. However, further research is needed to test this hypothesis.

It is also important to note that the risk factors identified in this study for DFD-related readmissions were comparable to those for all-cause readmissions, but the effect sizes, as well as confidence intervals, were generally larger for DFD-related readmissions. It is possible that this readmission group represents patients who presented with more complex DFD or were more susceptible to DFD-related complications due predisposition to unmeasured risk factors.

The current study has several limitations which must be considered. While administrative hospital admission data are acknowledged to under-report outcomes, including in-patients receiving care for DFD [55], it is important to acknowledge that in NSW, the quality of coded administrative data is monitored, validated, and subject to audit. This process enhances the reliability of the data used for analysis and consequently the validity of the study results. There is potential for response bias, as survey respondents were more likely to be older and male compared to the overall DFD admitted population. Additionally, since surveys were conducted three months after the hospitalisation experience, recall bias may have influenced responses. However, it is important to note that our findings are consistent with results from other studies conducted on patients with chronic conditions such as CHF and COPD [19], suggesting that any potential bias may not have affected the validity of our results. Despite our cohort including over 3000 adults, the proportion of respondents who expressed the most unfavourable feedback was relatively small, especially when considering DFD-related readmissions. In some instances, this limited the statistical power of the study to detect differences between the most and least favourable responses (e.g., 2% of patients rating the nurses or doctors who treated them as ‘poor’). Finally, whilst the multivariable shared-frailty Cox regression analysis considered important health and socio-economic determinants, there were several factors not adjusted for in our analysis, such as ulcer severity and post-discharge self-care compliance, that could influence readmission rates.

The current study provides insight into the quality of in-hospital patient experiences among persons admitted with DFD in NSW. For most patient experience indicators, there was a significant ranked relationship between patient reported measures and readmission, where the degree to which an experience measure is suboptimal increases the risk of DFD-patients returning to hospital for both DFD-related and all-cause readmissions within 90-days. Together, these findings can provide useful guidance for healthcare providers, hospital administrators, and policymakers to reduce DFD readmission rates. Potential interventions to improve the experiences of patients admitted for DFD should focus on enhancing health professional to patient communications, assessing patient readiness for discharge, and improving overall coordination of care.

## Supporting information

S1 Appendix (DOCX)

S2 Appendix (DOCX)
