# Supplementary material for: Patient reported experiences and readmissions for people with diabetes-related foot disease admitted to public hospitals, New South Wales, Australia, 2019–2022
Source: PLoS One. 2024 Dec 5;19(12):e0314895. doi: 10.1371/journal.pone.0314895 (PMC11620797; doi:10.1371/journal.pone.0314895)
Supplement: S1 Appendix — (DOCX) [file pone.0314895.s001.docx]

## **S1 Appendix**

The patient cohort of diabetic foot disease was defined using the Leading Better Value Care High Risk Foot Service Group 3: diabetic foot ulcers/infections and Group 4: diabetic foot procedures cohort definitions. These definitions were developed by the NSW Agency for Clinical Innovation and have been published elsewhere^[[1]](#footnote-2)^^[[2]](#footnote-3)^.

Diabetic foot disease hospital admissions were identified using the International Classification of Diseases and Related Health Problems, Tenth Revision, Australian Modification (ICD-10-AM) diagnoses codes and Australian Classification of Health Interventions (ICD-10-ACHI) procedure codes. ICD-10-AM codes include the principal or the first 50 secondary diagnoses and ICD-10-ACHI codes include the principal or the first 49 secondary procedures.

| Group 3: Diabetic foot ulcers/infections | Group 4: Diabetic foot procedures |
| --- | --- |
| Any of [E10, E11, E13, E14] (diabetes patients)  AND  any of [E10.73, E11.73, E13.73, E14.73, L03.02, L03.11, L03.13, L03.14, L97.x] (infection and/or ulcer), or [E10.51, E10.52, E11.51, E11.52, E13.51, E13.52, E14.51, E14.52] (peripheral vascular disease), or [E10.42, E11.42, E13.42, E14.42, E10.43, E11.43, E13.43, E14.43, E10.61, E11.61, E13.61, E14.61, E10.71, E11.71, E12.71, E13.71, E14.71] (peripheral neuropathy). | Any of [E10, E11, E13, E14] (diabetes patients)  AND  any of [32739-00, 3274200, 32745-00, 32748-00, 32751-00, 32751-01, 32751-02, 32754-00, 32754-01, 32754-02, 30023-00, 30023-01, 3022303, 32708-00, 32708-01, 32708-02, 32708-03, 32712-00, 32718-00, 32763-06, 32763-17, 33050-00, 33055-00, 3381804, 33818-05, 33818-06, 33818-07, 33821-05, 33821-06, 33821-07, 33848-00, 34172-00, 44338-00, 44358-00, 4436100, 44361-01, 44364-00, 44364-01, 44367-00, 44367-01, 44367-02, 44376-00, 45018-00, 45018-01, 45200-00, , 45206-10, 45206-11, 45221-00, 45224-00, 45227-00, 4523000, 45230-01, 45233-00, 45233-01, 45236-00, 45239-00, 45239-01, 45239-02, 45400-00, 45403-00, 45439-00, 4544200, 45445-00, 45448-10, 45448-11, 45451-09, 45451-26, 45451-27, 45496-00, 45497-00, 45498-00, 45499-00, 4556300, 45563-01, 45566-00, 45572-00, 48400-02, 48400-04, 48403-00, 48403-02, 48406-15, 48406-16, 48409-15, 48409-16, 49703-01, 49703-03, 49703-05, 49709-00, 49712-00, 49818-00, 50118-00, 90557-00, 90599-00, 9066500, 90669-00, 90672-00, 90686-01, 96210-00] (diabetic foot procedure) |

1. Deloitte Access Economics, Evaluation Plan and Evaluation Tools for the High Risk Foot Services Standards, Revised, NSW Agency for Clinical Innovation, November 2015, page 38. [↑](#footnote-ref-2)
2. Diabetes High Risk Foot Services, Monitoring and Evaluation Plan, NSW Agency for Clinical Innovation, Chatswood NSW, 2017, page 7. [↑](#footnote-ref-3)
