# Supplementary figures and images for: Patient reported experiences and readmissions for people with diabetes-related foot disease admitted to public hospitals, New South Wales, Australia, 2019–2022
Source: PLoS One. 2024 Dec 5;19(12):e0314895. doi: 10.1371/journal.pone.0314895 (PMC11620797; doi:10.1371/journal.pone.0314895)

## **S2 Appendix**


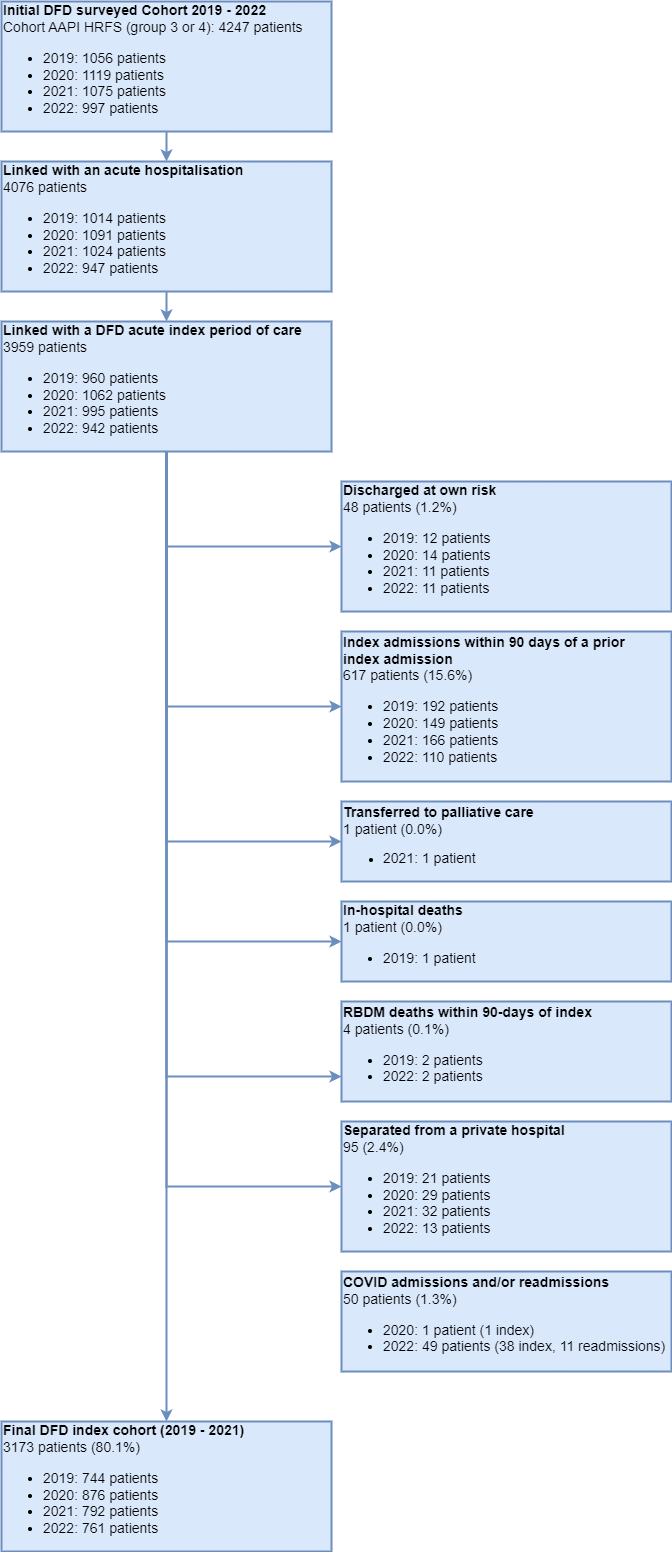

Supplement: S2 Appendix — (DOCX) [file pone.0314895.s002.docx]
